# Is Hydrogen Peroxide a Suitable Apoptosis Inducer for All Cell Types?

**DOI:** 10.1155/2016/7343965

**Published:** 2016-08-09

**Authors:** Jinmei Xiang, Chunyun Wan, Rui Guo, Dingzong Guo

**Affiliations:** ^1^College of Veterinary Medicine, Huazhong Agricultural University, Wuhan, Hubei 430070, China; ^2^Hubei Vocational College of Bio-Technology, Wuhan, Hubei 430070, China; ^3^College of Animal Science, Yangtze University, Jingzhou, Hubei 434023, China; ^4^Hubei Academy of Agricultural Sciences, Wuhan, Hubei 430070, China

## Abstract

Hydrogen peroxide is currently the most widely used apoptosis inducer due to its broad cytotoxic efficacy against nearly all cell types. However, equivalent cytotoxicity is achieved over a wide range of doses, although the reasons for this differential sensitivity are not always clear. In this study, three kinds of cells, the 293T cell line, primary fibroblasts, and terminally differentiated myocardial cells, were treated with a wide range of H_2_O_2_ doses. Times to apoptosis initiation and end were measured cytochemically and the changes in expression of caspase-9, P53, NF-*κ*B, and RIP were determined by RT-PCR. The 293T cell line was the most sensitive to H_2_O_2_, undergoing necroptosis and/or apoptosis at all concentrations from 0.1 to 1.6 mM. At > 0.4 mM, H_2_O_2_ also caused necroptosis in primary cells. At < 0.4 mM, however, primary cells exhibited classic signs of apoptosis, although they tended to survive for 36 hours in < 0.2 mM H_2_O_2_. Thus, H_2_O_2_ is a broadly effective apoptosis inducer, but the dose range differs by cell type. For cell lines, a low dose is required and the exposure time must be reduced compared to primary cells to avoid cell death primarily by necroptosis or necrosis.

## 1. Introduction

Cell apoptosis was first described as a cell death pathway unique from necrosis in 1972 [1]. Thereafter, a plethora of apoptosis inducers were identified, such as hydrogen peroxide (H_2_O_2_), dithiothreitol (DTT), and oxidized LDL [2–4]. Among these agents, H_2_O_2_ has been the most widely used and studied at the mechanistic level. In many cases, transient exposure to H_2_O_2_ triggers apoptosis through the mitochondrial pathway involving sequential loss of mitochondrial membrane potential, cytochrome c release, and effector caspase-3 activation [5–7]. Several factors that can antagonize apoptosis induced by H_2_O_2_ have also been identified, such as nerve growth factor (NGF) and chlorogenic acid [8, 9]. Hydrogen peroxide is used as an apoptosis inducer for many types of cells, including cell lines, tumor cells, primary cells, and highly differentiated cells [10–15], although the doses used vary widely, from 0.05 to 10 mM [12–14, 16, 17]. As there are several interacting but mechanistically distinct cell death pathways that may be activated by H_2_O_2_, it is critical to identify the ranges over which these pathways are primarily activated. Moreover, such information could yield valuable information on the interactions among these pathways under cell stress. To date, however, there is still no study that systematically studied different susceptibility to H_2_O_2_-induced apoptosis among cell types, which is critical for determining if H_2_O_2_ is a suitable apoptosis inducer in a specific context. Indeed, whether apoptosis or necroptosis is induced under different dosages of H_2_O_2_ is often unconfirmed [18] and apoptosis is only assumed.

Certain indices can reveal specific aspects of the cell death process. For example, caspase-9 can be used to monitor the initiation of apoptosis [19], activation of NF-*κ*B is usually caused by oxidative stress and may indicate DNA damage [20], and P53 can indicate dysregulation of the cell cycle and proliferation [21]. RIP is a key mediator of necroptosis signaling pathways; therefore RIP can be used to distinguish apoptosis from necroptosis [22].

To determine if H_2_O_2_ is a suitable apoptosis inducer for a given cell type, we measured cytochemical and genetic indices of apoptosis and necroptosis under a range of H_2_O_2_ doses in three cell types, an immortalized cell line, primary fibroblasts, and terminally differentiated cardiomyocytes. These cells were chosen for their distinct proliferative features. The 293T immortalized cell line is characterized by unlimited proliferation and passages, while fibroblasts show limited proliferation and passages, and terminally differentiated cardiomyocytes show no further proliferation. For convenience, both fibroblasts and myocardial cells were derived from chicken embryos.

## 2. Materials and Methods

### 2.1. Cell Culture

Isolation and culture of chicken embryo fibroblasts and myocardial cells followed methods previously described [23, 24] with some modifications. Briefly, White Leghorn eggs were obtained from Beijing Merial Vital Laboratory Animal Technology (Beijing, China). At embryonic day 11 (E11), embryos were removed and decapitated in a Petri dish filled with Medium 199/EBSS (HyClone, Logan, Utah, USA) supplemented with 3% fetal bovine serum (FBS, Gibco, Grand Island, New York, USA). Ventricular tissues and torso of chicken embryo were isolated for the preparations of myocardial cells and CEF and treated with 0.05% trypsin-EDTA to obtain a cell suspension as described [14, 25, 26], respectively. Specifically, the cells of CEF and myocardial cells were, respectively, incubated at 8 × 10^5^ per well in 24-well plates in growth medium (Medium 199/EBSS containing 10% FBS) at 37°C under a 5% CO_2_ atmosphere. Cultures were washed three times at 8, 24, and 48 h to remove dead and dying cells. The serum concentration in the medium was then changed from growth (10%) to maintenance (2%) conditions.

293T cells at low passage were incubated at 1 × 10^5^ cells/well in 24-well plates with Dulbecco's modified Eagle medium (DMEM) (HyClone, Logan, Utah, USA) containing 10% FBS at 37°C under a 5% CO_2_ atmosphere. After plating, the serum concentration was decreased from growth (10%) to maintenance (2%) conditions.

### 2.2. Apoptosis Induction and Assessment

Each cell type was divided into three groups, H_2_O_2_ treatment, positive control (DTT treatment), and negative control. H_2_O_2_ groups were incubated in 0.1, 0.2, 0.4, 0.8, or 1.6 mM H_2_O_2_, the positive control groups were incubated in 2 mM DTT, and the negative control groups received no treatment. After the start of exposure, cells were examined every 0.5 h by staining with AO/EB to monitor the initiation of apoptosis. DAPI staining was used to determine the time to substantial apoptosis. Then, apoptosis times and cell survival rates were determined by AO/EB staining and DAPI staining. Finally, total RNA was extracted from cells using Trizol reagent (Invitrogen, Carlsbad, CA, USA) to assess expression levels of apoptosis- and necroptosis-associated genes. Reverse transcription was performed using a PrimeScript II 1st Strand cDNA synthesis kit (TaKaRa, Otsu, Shiga, Japan). Quantitative real-time PCR was conducted to evaluate changes in caspase-9, P53, NF-*κ*B, and RIP expression levels using the primers and thermocycle conditions shown in Table 1. Group means were compared by ANOVA using SPSS17.0 software. All bar figures were created by Graphpad Prism 5 software.

## 3. Results

### 3.1. Initiation Time of Apoptosis

**Figure 1:**
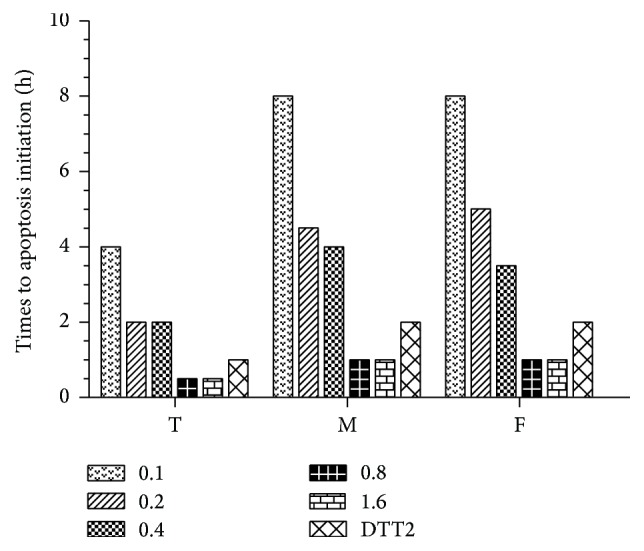
Time to apoptosis initiation. 293T cells (T), myocardial cells (M), and fibroblasts (F) were treated with a range of hydrogen peroxide concentrations (0.1–1.6 mM) or DTT (2 mM, positive control).

Apoptosis initiation times at different H_2_O_2_ doses are shown in Figure 1 for all three cell types. The time to initiation was estimated by AO/EB double staining every 0.5 h under an inverted epifluorescence microscope. The 293T cells were very sensitive to both DTT- and H_2_O_2_-induced apoptosis as indicated by the shorter delays until initiation compared to the other cell types. Alternatively, apoptosis initiation times did not differ significantly between myocardial cells and fibroblasts in response to DTT or H_2_O_2_.

### 3.2. Significant Differences in Times to Substantial Apoptosis and the End of Apoptosis among Cell Types

**Figure 2:**
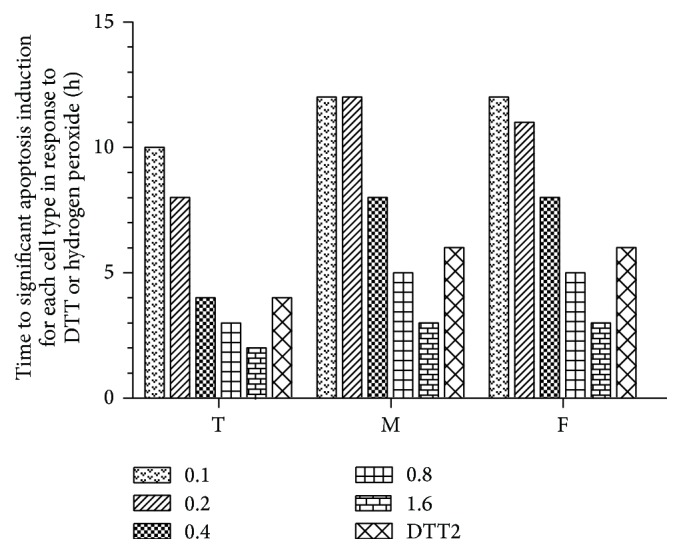
Time to significant apoptosis induction for each cell type in response to DTT or hydrogen peroxide. Hydrogen peroxide, 0.1–1.6 mM; DTT, 2 mM; T, 293T cells; M, myocardial cells; F, fibroblasts.

**Figure 3:**
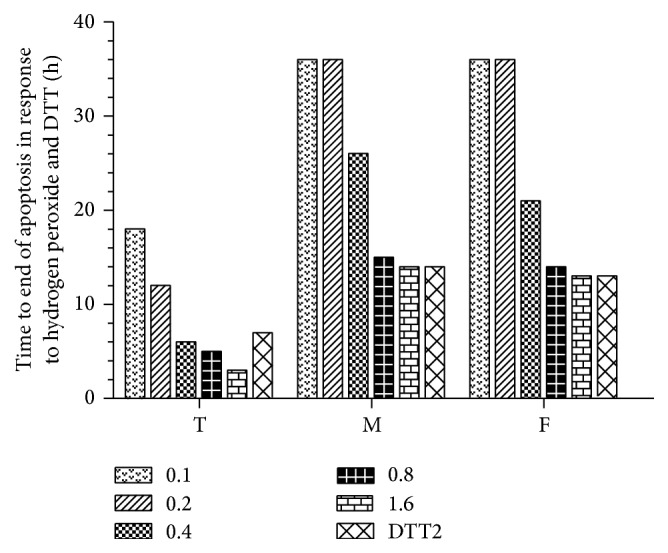
Time to end of apoptosis in response to hydrogen peroxide and DTT. Hydrogen peroxide, 0.1–1.6 mM; DTT, 2 mM; T, 293T cells; M, myocardial cells; F, fibroblasts.

After apoptosis started, DAPI staining was used to determine the time at which significant apoptosis occurred (Figure 2). At > 0.4 mM H_2_O_2_, apoptosis developed rapidly with little difference between cell types. At ≤ 0.4 mM, however, the time to substantial apoptosis was much more sensitive to H_2_O_2_ dose and cell type, with markedly faster times for 293T cells, particularly at 0.4 mM, compared to fibroblasts and cardiomyocytes. For the apoptosis ending test, the medium (both control and medium containing H_2_O_2_) was replaced every 12 hours to prevent a decrease in inducer concentration. Apoptosis ending was observed for up to 36 hours (Figure 3) and indicated that primary cells (fibroblasts and cardiomyocytes) can survive at 0.2 mM for 36 h.

### 3.3. Changes in Expression of Apoptosis Indicators

**Figure 4:**
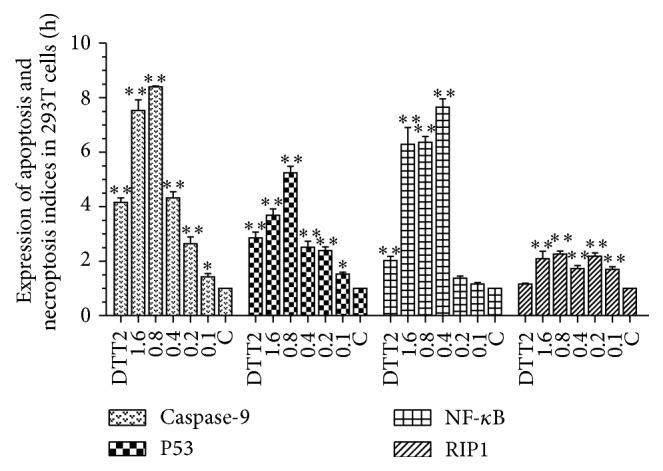
Expression of apoptosis and necroptosis indices in 293T cells. Hydrogen peroxide, 0.1–1.6 mM; DTT, 2 mM. Each bar represents the means ± SD (*n* = 3). ^*∗∗*^ *p* < 0.01; ^*∗*^ *p* < 0.05 compared with control.

**Figure 5:**
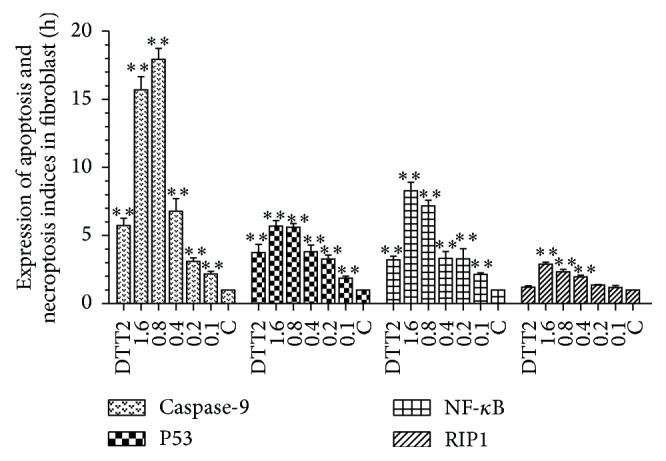
Expression of apoptosis and necroptosis indices in fibroblasts. Hydrogen peroxide, 0.1–1.6 mM; DTT, 2 mM. Each bar represents the means ± SD (*n* = 3). ^*∗∗*^ *p* < 0.01 compared with control.

**Figure 6:**
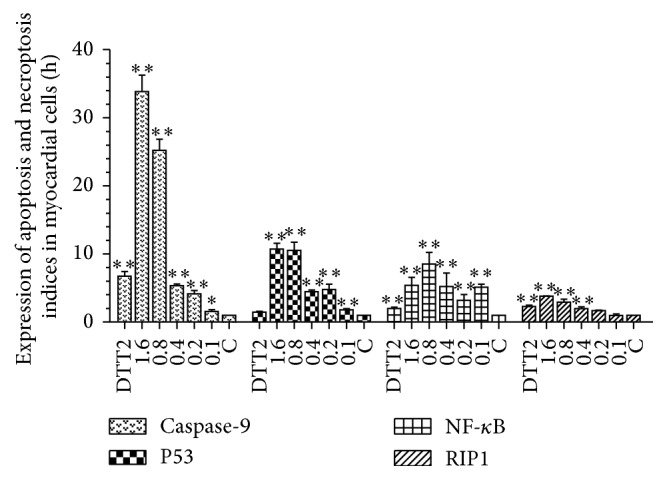
Expression of apoptosis and necroptosis indices in myocardial cells. Hydrogen peroxide, 0.1–1.6 mM; DTT, 2 mM. Each bar represents the means ± SD (*n* = 3). ^*∗∗*^ *p* < 0.01; ^*∗*^ *p* < 0.05 compared with control.

After apoptosis became significant, the RNA of each cell group was extracted and reverse-transcribed for real-time PCR analysis of caspase-9, P53, NF-*κ*B, and RIP expression levels. In 293T cells (Figure 4) caspase-9, P53, and NF-*κ*B increased at all H_2_O_2_ concentrations. In general, expression rose with increasing concentration but the peak differed for caspase-9, P53, and NK-*κ*B (0.6 or 0.8 mM). The trend for RIP was distinct; RIP was induced at all H_2_O_2_ concentrations, in contrast to the expression pattern in fibroblasts (Figure 5) and cardiomyocytes (Figure 6).

Hydrogen peroxide- and DTT-induced changes in fibroblast expression of apoptosis- and necroptosis-associated genes are shown in Figure 5. Compared to 293T cells, there were differences in the magnitude of the expression increases, but the general trends were similar. A notable exception was the higher threshold concentration for upregulation of the necroptosis indicator RIP.

Changes in expression also followed similar trends in myocardial cells (Figure 6) although NF-*κ*B expression was lower with no trend for dose-dependence. Moreover, similar to fibroblasts and in contrast to 293T cells, RIP expression was not upregulated by the lowest H_2_O_2_ concentrations.

## 4. Discussion

The three cell types examined showed clear differences in sensitivity to apoptosis induction by hydrogen peroxide and DTT. We speculated that highly proliferative cells would show the strongest apoptosis resistance, followed by primary cells and then highly differentiated cells. However, the exact opposite proved to be the case, with 293T cells showing the fastest apoptosis induction across H_2_O_2_ concentrations, as well as the shortest time to substantial induction, and the earliest end of induction compared to fibroblasts and cardiomyocytes. Moreover, the induction of apoptosis indices was accompanied by upregulation of RIP, a gene associated with necroptosis, at all H_2_O_2_ concentrations. Alternatively, terminally differentiated myocytes with no proliferative capacity showed minimal induction of apoptosis at concentrations inducing substantial apoptosis/necroptosis in 293T cells. In addition, internal ROS production may be different from one cell type to another and H_2_O_2_ produced during normal cell metabolism and production must be higher in rapidly proliferative cells. Therefore, H_2_O_2_ concentration used should be chosen carefully according to cell model in studies of apoptosis as the induction range differs markedly among cell types.

The necroptosis index RIP was induced by low concentrations of H_2_O_2_ (0.1 and 0.2 mM) only in 293T cells, while RIP induction required 0.4 mM or higher in fibroblasts and cardiomyocytes. Thus, H_2_O_2_ concentrations of 0.2–0.4 mM are appropriate for studying “pure” apoptosis in fibroblasts and cardiomyocytes. In contrast, very low doses may be required to study “pure” apoptosis in 293T cells. Therefore, if an inadequate dosage was used for specific cell types, the apoptosis inducing effect of H_2_O_2_ would not be observed and the appropriate dosage for inducing the apoptosis of specific cell types should be determined firstly. The series of measurements conducted here constitute a template for determining the optimal H_2_O_2_ concentration range for specific analysis of apoptosis (i.e., in the absence of necrosis or necroptosis) for a given cell type.

### 4.1. Efficiency of Hydrogen Peroxide

In our experiments, a hydrogen peroxide dosage was > 0.4 mM evoked rapid and relatively uniform cell death, while the extent of cell death was highly dose-sensitive ≤ 0.4 mM. At 0.1 and 0.2 mM, fibroblasts and cardiomyocytes survived for 36 hours, while substantial death of 293T cells was observed. Cell lines have a higher cell death efficiency induced by hydrogen peroxide. For all the cells, the starting time of apoptosis was dose-dependent; the higher dosage inducer had a faster cell death. In this experiment, we first wanted to use flow cytometry for assessment, but there were some interferences, especially in primary cells, for dregs can be easily stained and thus interfere with the assessment. Therefore, we used a fluorescence inversion microscope system.

### 4.2. Factors Controlling Susceptibility to Hydrogen Peroxide

It was found that cell density in a 24-well plate and the generation number of 293T cells has a significant impact on the beginning and ending times of apoptosis. If the cell line has a high generation or density, it would be more sensitive to the induction by hydrogen peroxide. So we decreased the number of the three kinds of cells per well during the incubation. The beginning and ending times of apoptosis were used to determine cell susceptibility to hydrogen peroxide. Unexpectedly, it was found that cell lines are the most sensitive to hydrogen peroxide. Even at the lower dosage, hydrogen peroxide may cause the apoptosis of 293T cells, and apoptosis can occur earlier and faster than the other 2 kinds of cells.

### 4.3. Cell Death Type after Induction with Hydrogen Peroxide

In the experiments, 4 indices were used to monitor the changes in cells after induction with hydrogen peroxide. As judged by RIP, it was found that 293T cells may be involved in necroptosis, even at a low dosage. Thus, if we want to induce cell lines into apoptosis, we must reduce the action time or use DTT to induce apoptosis. For myocardial cells and fibroblasts, they tend to be involved in necroptosis only at a higher dosage. From the other 3 indices, it can be concluded that 3 kinds of cells have different responses to the induction by hydrogen peroxide, which showed that fibroblasts are not inclined to necroptosis after induction from the expression of RIP. For other indices, fibroblasts have a higher expression of NF-*κ*B, which may indicate that it has had more DNA corrosion and handicap of transcription. 293T cells have a higher expression of P53, indicating a disorder in cell cycle and proliferation. The myocardial cells have a higher expression of all indices, which may be a comprehensive effect.

In the experiments it was found that the cells underwent morphologic changes after induction by hydrogen peroxide and it is dose-dependent. After the comparison of DTT and hydrogen peroxide, there are many differences in the 4 indices; however, the trend is basically consistent. It was also found that the hydrogen peroxide can greatly influence cell motor ability, but after the induction of DTT for 6 hours, the impulse of some myocardial cells still exists and the apoptosis effect is less than hydrogen peroxide. The existing question is whether or not hydrogen peroxide has a selectivity in different cell lines or if hydrogen peroxide has a species selectivity, such as cells from rats.

## 5. Conclusion

Hydrogen peroxide has a high efficiency leading to cell death. Hydrogen peroxide causes necroptosis in 293T cells at a concentration ranging from 0.1 to 1.6 mM. The cell lines used in this study were sensitive to hydrogen peroxide. In primary cells, a concentration > 0.4 mM may also cause necroptosis. A concentration < 0.4 mM had a tendency to apoptosis. Primary cells can survive in hydrogen peroxide for 36 hours at a concentration ≤ 0.2 mM. Different cells have a different response to induction by hydrogen peroxide. Thus, hydrogen peroxide qualifies as an apoptosis inducer at a specific dosage corresponding to the specific cell types, but researchers do not pay attention to these findings.

## Supplementary Material

Figure S1. Culture and identification of cells. Figures showed the morphology character of the normal and apoptosis-induced in myocardial cells. A showed the myocardial cells that cultured for 24 h. B showed the identification of myocardial cells. The myocardial cells were identified by cellular immunohistochemistry and detected the α-actin antigen by DAB staining. C to H showed that the cells were in apoptosis-induced and stained by AO/EB or DAPI respectively.

## Figures and Tables

**Table 1:** Primers for quantitative real-time PCR.

	Gene	Forward	Reverse	Thermocycle
F/C	Caspase-9	5′TCAGACATCGTATCCTCCA	5′AGTCACAGCAGCACA	98°C/3 min + 40 × [98°/15 s; 61°C/40 s]
	P53	5′ACCTGCACTTACTCCCCGGT	5′TCTTATAGACGGCCACGGCG	
	NF-*κ*B	5′AGGACTTAAAATGGCAGGAGAG	5′GCTGTTCGTAGTGGTAAGTCTG	
	RIP1	5′GAACTAGGCTTCAGCAACTCCG	5′GCAGCCAAAGAGGGCTTTGG	
	*β*-actin	5′GGCACCCAGCACAATGAAG	5′CCGATCCACACGGAGTACTTG	

293T	Caspase-9	5′TGCTGAGCAGCGAGCTGTT	5′AGCCTGCCCGCTGGAT	98°C/3 min + 40 × [98°/15 s; 61°C/40 s]
	P53	5′CCACCATCCACTACAACTACAT	5′CAAACACGGACAGGACCC	
	NF-*κ*B	5′TCTCCCTGGTCACCAAGGAC	5′TCATAGAAGCCATCCCGGC	
	RIP1	5′CATGGAAAAGGCGTGATACAC	5′ACTTCCCTCAGCTCATTGTG	
	*β*-actin	5′GGCACCCAGCACAATGAAG	5′CCGATCCACACGGAGTACTTG	

F/C: fibroblasts/cardiomyocytes.
