# Supplementary material for: Is Hydrogen Peroxide a Suitable Apoptosis Inducer for All Cell Types?
Source: Biomed Res Int. 2016 Aug 9;2016:7343965. doi: 10.1155/2016/7343965 (PMC4993923; doi:10.1155/2016/7343965)
Supplement: Supplementary file 1 — Figure S1. Culture and identification of cells. Figures showed the morphology character of the normal and apoptosis-induced in myocardial cells. A showed the myocardial cells that cultured for 24 h. B showed the identification of myocardial cells. The myocardial cells were identified by cellular immunohistochemistry and detected the α-actin antigen by DAB staining. C to H showed that the cells were in apoptosis-induced and stained by AO/EB or DAPI respectively. [file 7343965.f1.pdf]

Supporting information

Figure S1. Culture and identification of cells. (A) The cell morphology of myocardial cells. (B) The cell identification by cellular immunohistochemistry and detection of a-actin antigen by DAB. (C) cells are shown to have significant morphologic changes after induction of apoptosis (0.2 mM, 4 h). (D) The normal AO/EB staining. (E) The beginning of apoptosis by DAPI. (F) The significant apoptosis in cells by AO/EB staining. (G) and (H) completion of apoptosis by AO/EB staining, respectively.

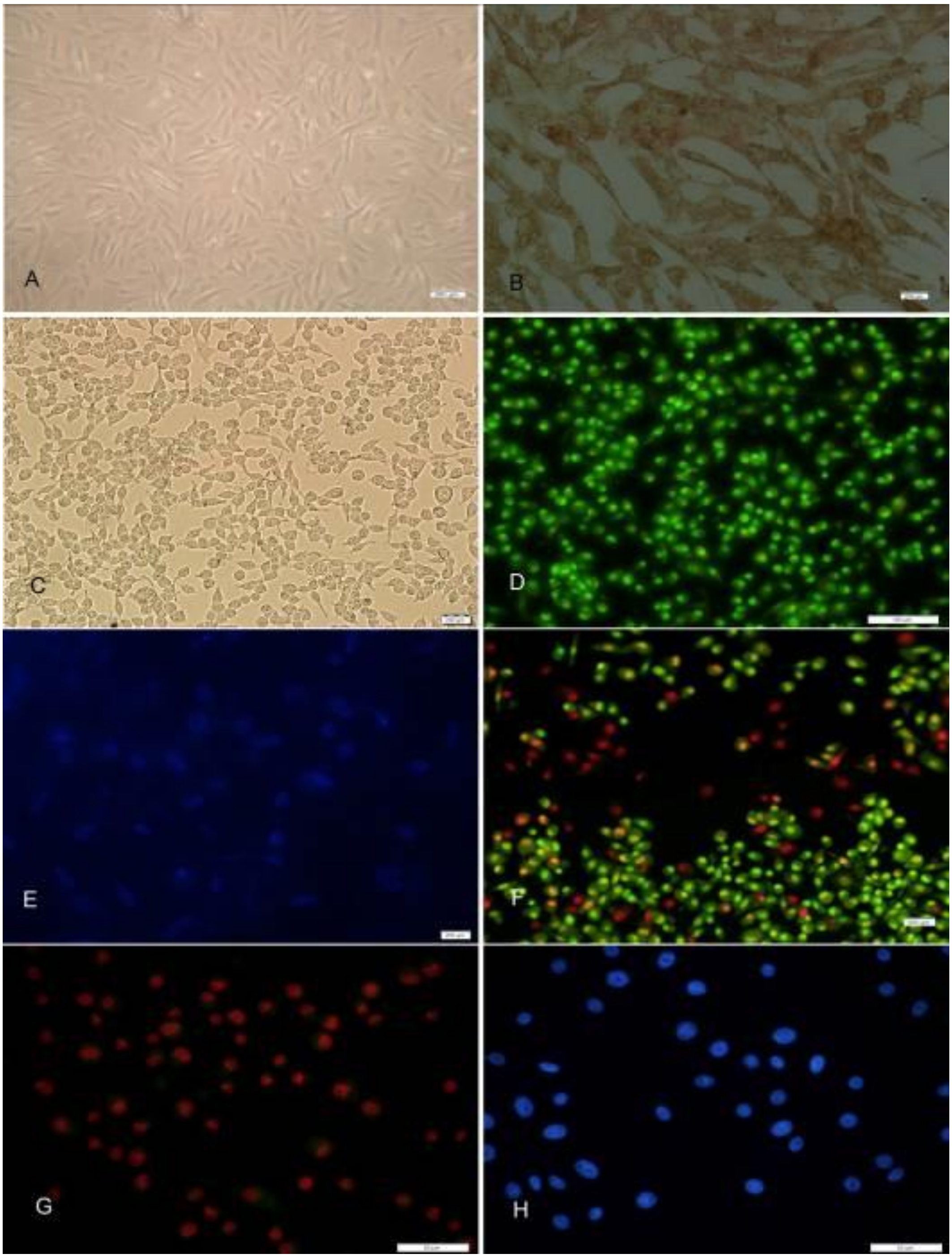
A
B
C
D
E
F
G
H
